# An oncolytic adenovirus that expresses the HAb18 and interleukin 24 genes exhibits enhanced antitumor activity in hepatocellular carcinoma cells

**DOI:** 10.18632/oncotarget.11134

**Published:** 2016-08-09

**Authors:** Sujing Yuan, Xianlong Fang, Yanni Xu, Aimin Ni, Xin-Yuan Liu, Liang Chu

**Affiliations:** ^1^ State Key Laboratory of Cell Biology, Institute of Biochemistry and Cell Biology, Shanghai Institutes for Biological Sciences, Chinese Academy of Sciences, Shanghai 200031, P. R. China; ^2^ College of Life Sciences, Northwest Agriculture and Forestry University, Yangling 712100, P. R. China; ^3^ Jiangsu Center for the Collaboration and Innovation of Cancer Biotherapy, Cancer Institute, Xuzhou Medical College, Xuzhou 221002, P. R. China

**Keywords:** oncolytic adenovirus, HAb18, CD147, interleukin 24, hepatocellular carcinoma

## Abstract

Hepatocellular carcinoma (HCC) is characterized by alterations in multiple genes. High expression of CD147 on the surface of HCC cells promotes proliferation. The monoclonal antibody HAb18 recognizes CD147. We constructed an oncolytic adenoviral vector to express HAb18 (ZD55-HAb18) in HCC cells. Interleukin 24 (IL24) was co-expressed through the use of an F2A linker. ZD55-HAb18-IL24 decreased HCC cell viability to a greater degree than either ZD55-HAb18 or ZD55-IL24 alone. ZD55-HAb18-IL24 also induced apoptosis and autophagy in PLC/PRF/5 HCC cells. Intratumoral injection of ZD55-HAb18-IL24 repressed tumor growth in a PLC/PRF/5 xenograft model. Our results suggest that antibody-antitumor gene conjugation elicited a stronger antitumor effect than the antibody alone, and that this strategy could broaden the applications of antibody-based therapies in HCC.

## INTRODUCTION

Hepatocellular carcinoma (HCC) is a multi-factorial disease that involves cross-talk between several pathways [1]. Combination approaches for HCC therapy based on multi-targeted conjugates have significant advantages [2]. Combination endostatin/sFlt-1 antiangiogenic gene therapy was shown to be highly effective in a rat model of HCC [3]. Complete eradication of HCC was achieved by combined vasostatin gene therapy and B7H3-mediated immunotherapy [4]. Drozdzik et al. demonstrated that a suicide gene in combination with interleukin-12 was more efficient than therapy with one gene alone in a murine model of HCC [5].

CD147 is a transmembrane glycoprotein that belongs to the immunoglobulin superfamily and is highly enriched on the surface of human HCC cells [6]. CD147 plays a critical role in tumor progression and metastasis [7]. Recent studies have revealed that knockdown of CD147 reduced cell proliferation and improved chemo-sensitivity in many cancer cells [8, 9]. HAb18 is a single-chain monoclonal antibody fragment (scFv). The variable regions of the heavy chain and light chains are linked by a (GGGS)_3_ peptide. HAb18 is secreted by hybridoma cells in BALB/c mice immunized with human HCC tissue extracts. The antigen recognized by HAb18 is CD147, which was identified in a screen of an HCC cDNA library [10-12]. Xu et al. reported that blocking CD147 with HAb18 mAb inhibited HCC growth and metastasis *in vivo* [13].

Interleukin 24 (IL24) is a cytokine that belongs to the IL-10 family of cytokines [14]. Preclinical studies have shown that ectopic expression of IL24 induces apoptosis in cancer cells with no significant cytotoxicity to normal cells [15, 16]. IL24 has synergistic effects in various human cancers when combined with other agents. For example, Ad-IL24 combined with a selective inhibitor of EGFR (gefitnib) induces apoptotic cell death in non-small cell lung cancer [17]. In another study, treatment of Her-2/neu-overexpressing breast cancer cells with Ad-IL24 in combination with a monoclonal antibody targeting the Her-2/neu receptor inhibited cell growth [18]. These studies indicate therapeutic antibodies combined with IL24 might enhance antitumor efficacy.

Oncolytic adenoviral vectors are promising cancer therapies [19-21]. The ONYX-015 vector was engineered to lack expression of the E1B55KD viral protein. Reduced replication of ONYX-015 in normal cells results from defective export of late viral RNA. This is because the E1B55KD protein facilitates preferential transport of viral RNA during the late stages of oncolytic adenoviral infection [22, 23]. We constructed the oncolytic adenovirus ZD55, in which the E1B55KD gene was deleted and a cloning site added in order to insert foreign antitumor genes [24]. Previous studies have shown that ZD55 carrying the IL24 gene could selectively replicate in tumor cells and inhibit cell growth more effectively than ONYX-015 and a replication-defective adenovirus carrying the IL24 gene [25]. In another study, a combination of ZD55 carrying the TRAIL gene and ZD55 carrying the Smac gene suppressed the growth of HCC tumors in mice [26].

In this study, we used ZD55 to express HAb18-IL24 linked by a foot-and-mouth-disease virus (FMDV)-derived 2A self-processing peptide (F2A). ZD55-HAb18-IL24 decreased HCC cell viability, induced apoptosis and autophagy, and inhibited tumor growth in a PLC/PRF/5 xenograft model. Additionally, ZD55-HAb18-IL24 displayed potent antiangiogenic activity *in vivo*. Our results indicate oncolytic adenoviruses carrying a combination of a therapeutic monoclonal antibody and a cytokine may be effective HCC therapies.

## RESULTS

### CD147 is highly expressed in HCC and promotes cell proliferation and chemoresistance

**Figure 1:**
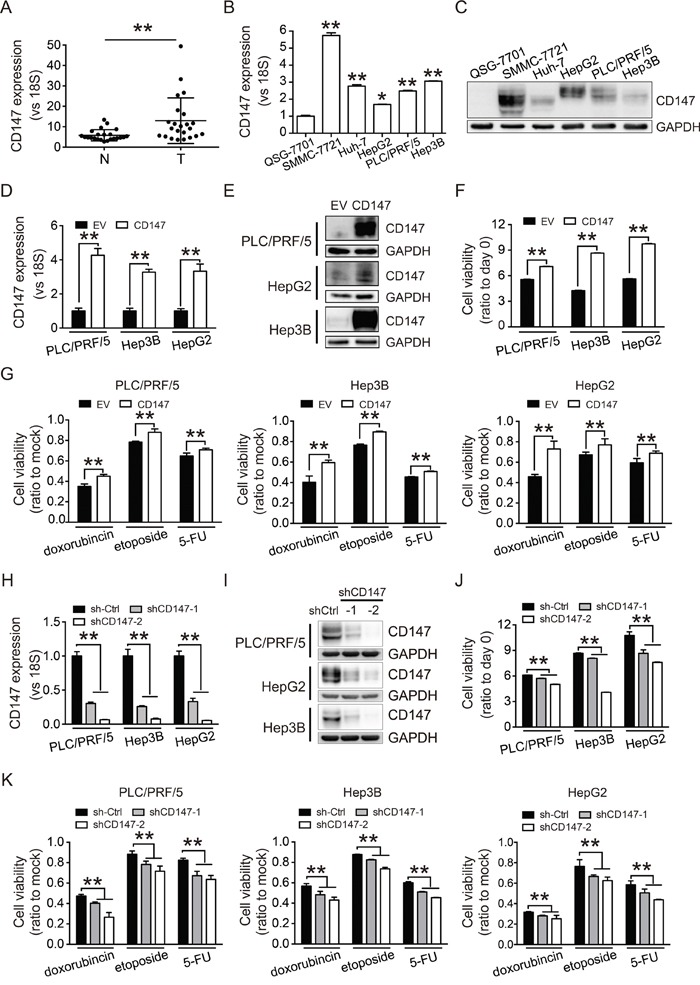
CD147 is highly expressed in HCC and promotes cell proliferation and chemoresistance **A.** Levels of CD147 mRNA in 23 HCC tissue (T) and adjacent non-cancerous tissue (N) were measured by qRT-PCR. **B, C.** Expression of CD147 in HCC cell lines (SMMC-7721, Huh-7, HepG2, PLC/PRF/5, and Hep3B) and normal hepatocytes (QSG-7701) was measured by qRT-PCR (B) and western blot (C). **D, E.** CD147 overexpression was confirmed by qRT-PCR (D) and western blot (E). **F.** CD147 overexpression increases proliferation in PLC/PRF/5, Hep3B, and HepG2 cells. **G.** CD147 overexpression increases the resistance of PLC/PRF/5, Hep3B, and HepG2 cells to cytotoxic chemotherapy. **H, I.** CD147 knockdown was confirmed by qRT-PCR (H) and western blot (I). **J, K.** Knockdown of CD147 inhibits cell proliferation (J) and decreases resistance to cytotoxic chemotherapy (K). Cell proliferation was measured using CCK-8 assays at 96 (F, J) and 48 hours (G, K). Cells were treated with doxorubicin (1 μg/mL), etoposide (10 μg/mL), and fluorouracil (5-FU; 100 μg/mL) for 2 days. Relative cell viability is shown as the fold change compared to mock control cells (G, K). GAPDH was used as a loading control in (C), (E), and (I). The qRT-PCR data in (A), (B), (D), and (H) were normalized to 18S RNA and are shown as the fold change relative to QSG-7701 cells in (B), EV groups in (D), and sh-Ctrl groups in (H). EV, empty vector. Sh-Ctrl, scramble shRNA vector control. All experiments were repeated three times. The bars represent the mean ± S.D. (n = 3), *p < 0.05, **p < 0.01.

CD147 expression in HCC tissue (T) and adjacent non-cancerous tissue (N) was evaluated by quantitative real-time PCR (qRT-PCR) (Figure 1A). CD147 was found to be overexpressed in tumor compared to normal tissue. Examination of CD147 expression in several HCC cell lines revealed higher CD147 mRNA and protein expression in cancer cells compared to normal human hepatocytes (QSG-7701 cells) (Figure 1B and 1C).

To investigate the effects of CD147 on HCC cell proliferation, we overexpressed CD147 in PLC/PRF/5, Hep3B, and HepG2 cells using a lentiviral infection strategy. Cells that stably overexpressed CD147 were selected with puromycin (Figure 1D and 1E). Overexpression of CD147 promoted cell proliferation (Figure 1F) and conferred resistance to cytotoxic chemotherapy (Figure 1G). We next knocked down CD147 using lentivirus-mediated shRNA (Figure 1H and 1I). Knockdown of CD147 inhibited cell growth (Figure 1J) and sensitized the cells to chemotherapy (Figure 1K). These results demonstrated that CD147 enhanced cell proliferation and protected cells from the effects of chemotherapy, suggesting that it might be an attractive therapeutic target in HCC.

### ZD55-HAb18-IL24 exhibits enhanced cytotoxicity *in vitro*

**Figure 2:**
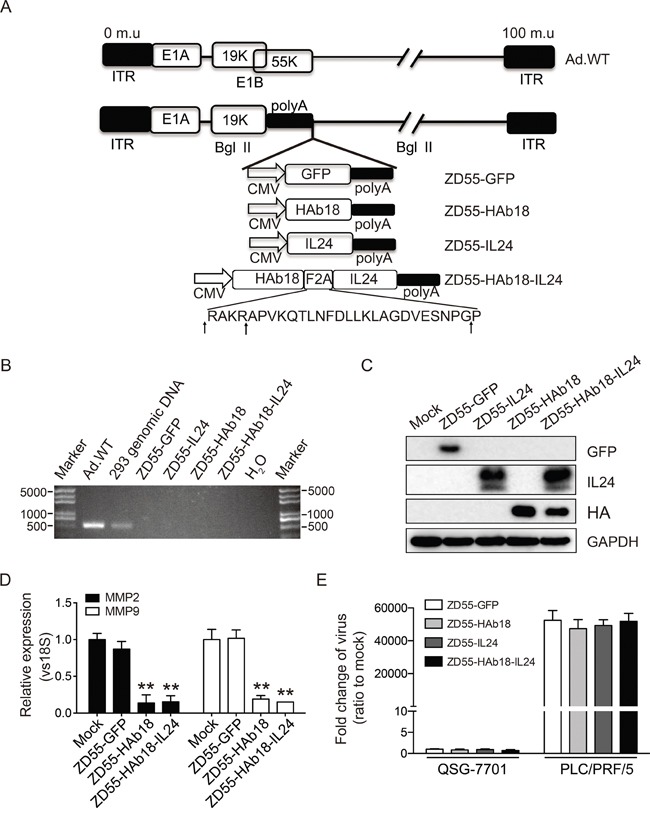
Construction and characterization of the oncolytic adenoviruses **A.** Schematic of the structures of the oncolytic adenoviruses. The amino acid sequence of F2A is shown. Arrows indicate the cleavage sites in F2A. Ad.WT, wild-type adenovirus. ITR, inverted terminal repeat. **B.** Characterization of oncolytic adenoviruses by PCR using the E1B-55KD forward and reverse primers (Supplementary Table S1). All oncolytic adenovirus showed no wild-type contamination. **C.** Western blot detection of genes carried by ZD55 in PLC/PRF/5 cells 48 hours after oncolytic adenovirus infection (10 MOI). GAPDH was used as a loading control. **D.** Analysis of MMP-2 and MMP-9 in PLC/PRF/5 cells treated with the indicated oncolytic adenoviruses (MOI of 10) for 48 hours by qRT-PCR. **E.** Analysis of the replication ability of the oncolytic adenoviruses in normal hepatocytes (QSG-7701) and tumor cells (PLC/PRF/5) by qRT-PCR. The adenovirus E1A region was amplified to evaluate viral replication 48 hours after infection (MOI of 10). The qRT-PCR data in (D) and (E) were normalized to 18S, and are shown as the fold change relative to mock cells. All experiments were repeated three times. The bars represented the mean ± S.D. (n = 3). **p < 0.01.

The effects of CD147 prompted us to assess whether inhibition of CD147 with the monoclonal antibody HAb18 could have therapeutic efficacy. We also simultaneously introduced IL24 into the oncolytic adenoviral vector since multi-target agents have been widely investigated for cancer treatment. We hypothesized that a combination of HAb18 and IL24 could improve the therapeutic efficacy. To test this hypothesis, we constructed an oncolytic adenovirus that expressed the HAb18 (ZD55-HAb18) and IL24 (ZD55-IL24) genes. We linked the two genes with an F2A sequence and cloned the product into an oncolytic adenoviral vector to generate ZD55-HAb18-IL24 (Figure 2A). PCR analysis of the E1B-55KD gene confirmed that all the oncolytic adenoviruses were free of wild-type adenovirus contamination (Figure 2B).

The expression of exogenous HAb18, IL24, and GFP in PLC/PRF/5 cells was detected by western blot (Figure 2C). PLC/PRF/5 cells infected with ZD55-HAb18-IL24 expressed HAb18 and IL24 individually with bands corresponding to the correct molecular weights. The expression of both proteins in ZD55-HAb18-IL24-treated cells was similar to expression in cells infected with either ZD55-HAb18 or ZD55-IL24 individually, indicating that the F2A peptides mediated efficient generation of the individual HAb18 and IL24 proteins. We observed decreases in MMP-2 and MMP-9 expression in ZD55-HAb18- and ZD55-HAb18-IL24-treated PLC/PRF/5 cells, which suggested that oncolytic adenoviral expression of mAb HAb18 inhibited CD147 function (Figure 2D).

To compare the abilities of the oncolytic adenoviruses to replicate in tumor and normal cell lines, PLC/PRF/5 and QSG-7701 cells were infected with oncolytic adenoviruses at an MOI of 10. The relative replication efficacy of the oncolytic adenoviruses was measured by qRT-PCR of adenovirus gene E1A cDNA. The oncolytic adenoviruses replicated effectively in tumor cells (Figure 2E). In contrast, the replication ability was significantly reduced in normal cells. These results demonstrated that oncolytic adenoviruses selectively replicated in tumor cells.

**Figure 3:**
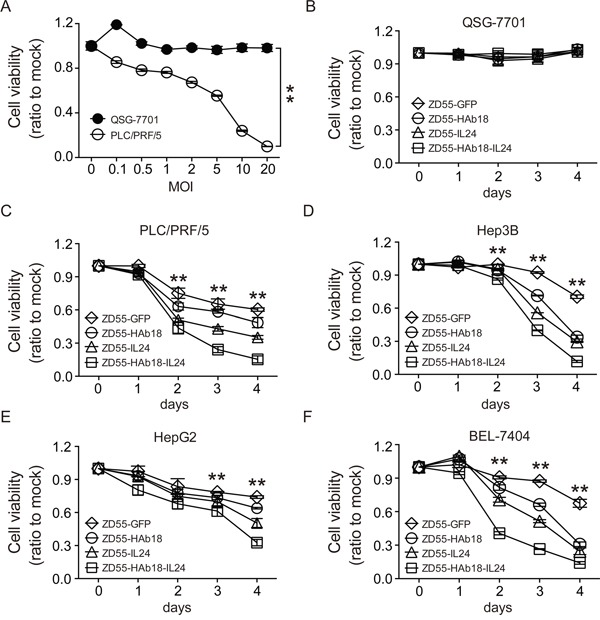
ZD55-HAb18-IL24 displays antitumor activity *in vitro* **A.** The viability of PLC/PRF/5 and QSG-7701 cells infected with ZD55-HAb18-IL24 at the indicated MOIs on day 4. **B-F.** The viability of QSG-7701, PLC/PRF/5, Hep3B, HepG2, and BEL-7404 cells infected with the indicated oncolytic adenoviruses for 4 days at an MOI of 10. Cell viability was assessed using CCK-8 assays. All experiments were repeated three times. The bars represent the mean ± S.D. (n = 3), *p < 0.05, **p < 0.01.

We next evaluated the cytotoxicity of ZD55-HAb18-IL24. PLC/PRF/5 HCC cells were infected with ZD55-HAb18-IL24 at a series of MOIs from 0.05–20. Cell viability was measured 4 days later and the IC50 of ZD55-HAb18-IL24 determined. ZD55-HAb18-IL24 significantly inhibited PLC/PRF/5 cell growth (IC50 = 9.21 ± 1.24 MOI). Importantly, a rare cytopathic effect of ZD55-HAb18-IL24 was observed in QSG-7701 cells (even at an MOI of 20), suggesting that ZD55-HAb18-IL24 was cytotoxic to cancer cells but not normal cells (Figure 3A). The oncolytic adenoviruses had no effect on the growth of QSG-7701 cells transduced with CD147-expressing lentiviruses (Supplementary Figure S1). We next compared the antitumor effects of ZD55-HAb18-IL24, ZD55-HAb18, and ZD55-IL24. Cells were infected with the indicated oncolytic adenoviruses at an MOI of 10 for 4 days. ZD55-HAb18-IL24 inhibited HCC cell growth to a greater extent than either ZD55-HAb18 or ZD55-IL24 alone (Figure 3B-3F), indicating ZD55-HAb18-IL24 had higher antitumor efficacy.

### ZD55-HAb18-IL24 induces apoptosis and autophagy

**Figure 4:**
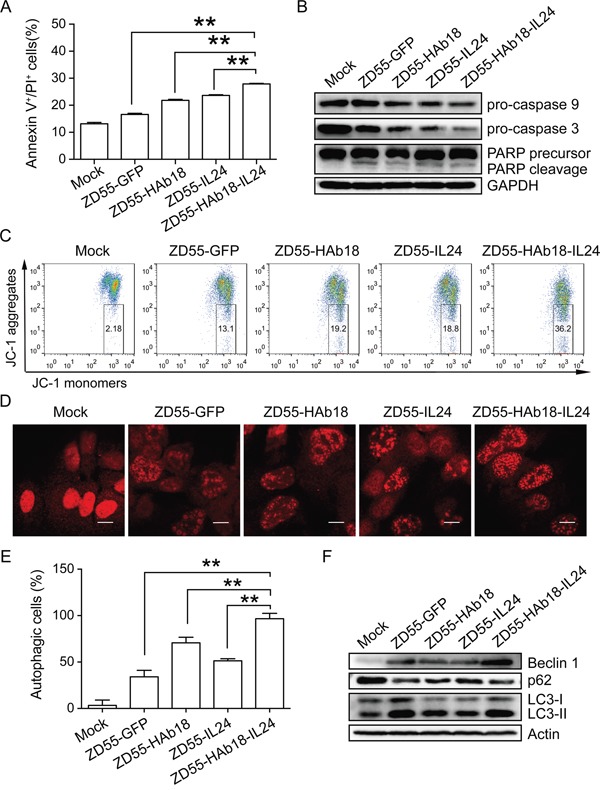
ZD55-HAb18-IL24 induces apoptosis and autophagy in PLC/PRF/5 cells **A.** Apoptosis was evaluated in oncolytic adenovirus-infected PLC/PRF/5 cells using Annexin V/PI staining. **B.** Western blot detection of procaspase-9, procaspase-3, and PARP in PLC/PRF/5 cells. GAPDH was used as a loading control. **C.** Oncolytic adenoviruses alter the mitochondrial membrane potential (Δψm) in PLC/PRF/5 cells. The numbers in the quadrilateral region show the ratio of cells with altered Δψm. **D.** Representative images of LC3 puncta in PLC/PRF/5 cells infected with indicated oncolytic adenoviruses. Scale bars, 10 μm. **E.** Statistical data for the three repeats in (D). **F.** Western blot detection of beclin-1, p62, and LC3-I/II in oncolytic adenovirus-infected PLC/PRF/5 cells. Actin was used as a loading control. PLC/PRF/5 cells were infected with the indicated oncolytic adenoviruses (MOI of 10) for 2 days. All experiments were repeated three times. The bars represent the mean ± S.D. (n = 3). **p < 0.01.

We assessed whether ZD55-HAb18-IL24 could induce apoptosis in PLC/PRF/5 cells 48 hours after oncolytic adenovirus infection using Annexin V/propidium iodide (PI) staining. The percentage of apoptotic cells (Annexin V^+^/PI^+^) in the ZD55-HAb18-IL24-treated group was higher than in the other groups (Figure 4A). We also detected several key members of caspase-dependent apoptotic signaling cascades by western blot. Enhanced poly ADP-ribose polymerase (PARP) cleavage, and decreased expression of procaspase-3 and procaspase-9 were observed in ZD55-HAb18-IL24-treated PLC/PRF/5 cells (Figure 4B). Because cleaved caspase-9 is an essential initiator of the mitochondrial apoptosis signaling pathway, we analyzed changes in the mitochondrial membrane potential (Δψm) using JC-1 staining. An increase in the number of cells with mitochondrial membrane potential loss was observed in ZD55-HAb18-IL24-treated PLC/PRF/5 cells (Figure 4C). These data suggested that ZD55-HAb18-IL24 induced apoptosis in PLC/PRF/5 cells through the mitochondrial pathway.

Previous data indicated CD147 could inhibit autophagy in cancer cells [27, 28]. Microtubule-associated protein light chain 3 (LC3) is essential for autophagosome formation and is an accurate autophagosome marker. ZD55-HAb18-IL24-treated PLC/PRF/5 cells exhibited a higher percentage of cells with LC3 puncta compared to the other oncolytic adenovirus-treated cells (Figure 4D and 4E). Increased expression of beclin-1 (an autophagy protein), degradation of p62 (an autophagy substrate), and conversion of the nonlipidated form of LC3 (LC3-I) to the phosphatidylethanolamine-conjugated form (LC3-II) were observed in ZD55-HAb18-IL24-treated cells (Figure 4F). These data demonstrated that ZD55-HAb18-IL24 induced autophagy in PLC/PRF/5 cells.

### ZD55-HAb18-IL24 suppresses tumor growth *in vivo*

**Figure 5:**
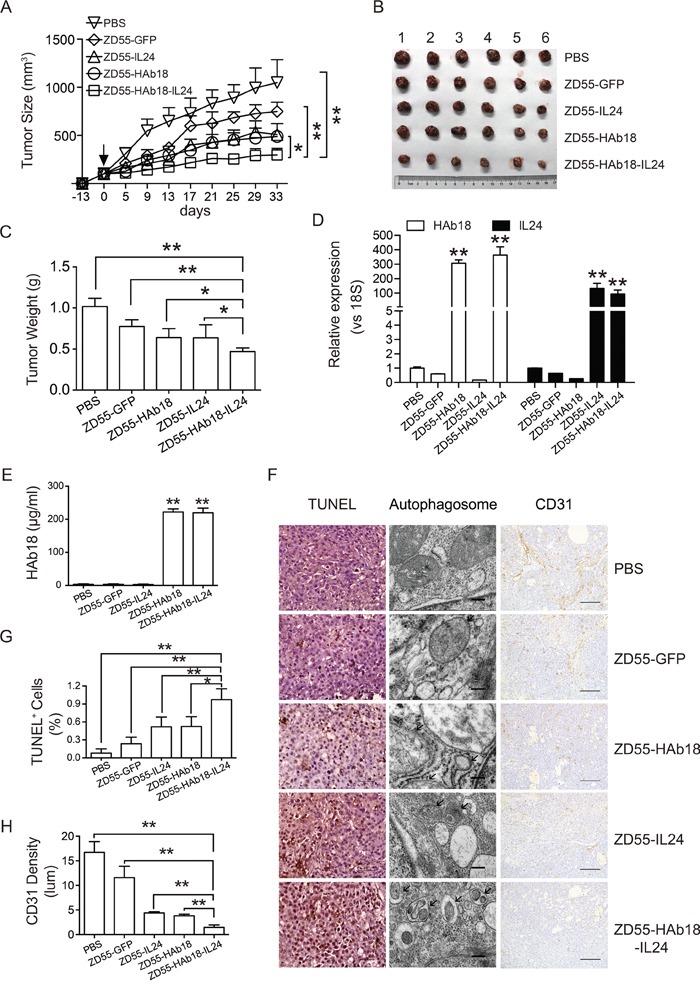
ZD55-HAb18-IL24 displays antitumor activity *in vivo* **A.** Growth curve of PLC/PRF/5 xenograft tumors after intratumoral injection of the indicated adenoviruses. Oncolytic adenoviruses were injected 13 days after subcutaneous inoculation of PLC/PRF/5 cells (day 0, arrow). The corresponding volumes of PBS were injected as a control. **B, C.** Tumors were excised, weighed, and photographed 33 days after injection. Significant decreases in tumor volume (B) and weight (C) were observed in the ZD55-HAb18-IL24-treated group. The bars represent the mean ± S.D. (n = 6). **D.** qRT-PCR detection of HAb18 and IL24 expression in tumors from each group. Data were normalized to 18S, and are shown as the fold change relative to PBS-treated cells. **E.** ELISA detection of HAb18 concentration in serum 33 days after virus injection. **F.** Representative images of TUNEL staining (scale bar, 100 μm; left panel), electron microscopy (scale bar, 200 nm; middle panel), and CD31 immunohistochemical staining (scale bar, 100 μm; right panel) of tumor sections from each group. **G.** Quantification of the TUNEL staining results in (F). **H.** Quantification of CD31-positive cells in (F). The bars represent the mean ± S.D. of triplicate samples. *p < 0.05, **p < 0.01.

To evaluate the therapeutic potential of oncolytic adenoviruses *in vivo*, oncolytic adenoviruses and corresponding volumes of PBS were intratumorally injected in PLC/PRF/5 xenografts. Treatment with ZD55-HAb18-IL24 delayed tumor growth compared to treatment with the other oncolytic adenoviruses (Figure 5A). The mice were sacrificed 33 days after injection. The tumor volume and weight in the ZD55-HAb18-IL24-treated group were 297.8 ± 67.5 mm^3^ and 47 ± 0.05 g, respectively. These tumors were significantly smaller than the tumors in the other groups (Figure 5B and 5C), indicating that ZD55-HAb18-IL24 was able to efficiently inhibit PLC/PRF/5 xenograft growth. HAb18 and IL24 expression in tumors was assessed by qRT-PCR, and the serum HAb18 level examined by enzyme-linked immunosorbent assays (ELISA) at the end of the experiment. ZD55-HAb18-IL24 expressed high levels of HAb18 and IL24 in tumors (Figure 5D). The expression of HAb18 in serum remained relatively high for > 1 month (Figure 5E). We analyzed the kinetics of HAb18 expression in nude mice bearing PLC/PRF/5 tumors. These results demonstrated that ZD55-HAb18 and ZD55-HAb18-IL24 efficiently expressed HAb18 (Supplementary Figure S2). Additionally, no significant increases in the expression of aspartate aminotransferase (AST), alanine aminotransferase (ALT), blood urea nitrogen (BUN), or creatinine were observed after treatment (Supplementary Figure S3).

The apoptotic and autophagic effects of ZD55-HAb18-IL24 in PLC/PRF/5 xenografts were analyzed using terminal deoxynucleotidyl transferase dUTP nick end labeling (TUNEL) assays. A higher percentage of positive cells was observed in the ZD55-HAb18-IL24-treated tumors than in the other groups (Figure 5F and 5G). Tumors in the ZD55-HAb18-IL24-treated groups displayed more autophagic structures than tumors in the other groups, and no autophagic structures were observed in PBS-treated tumors by electron microscopy (Figure 5F). Because angiogenesis plays a critical role in tumor progression, we examined the expression of VEGFA in adenovirus-treated cells *in vitro* and *in vivo*. A significant decrease in VEGFA expression was observed after adenovirus infection (Supplementary Figure S4). Immunohistochemical staining of CD31, a vascular endothelial marker, revealed reduced microvessel density in ZD55-HAb18-IL24-treated tumors (Figure 5F and 5H).

## DISCUSSION

HCC can result from both genetic and epigenetic alterations [29]. Many factors involved in HCC development are potential therapeutic targets. CD147 is an HCC-associated antigen and plays important roles in HCC progression [30]. Our data indicate that CD147 promotes HCC cell proliferation and chemoresistance (Figure 1). Overexpression of CD147 was also shown to promote proliferation and chemoresistance in bladder cancer T24 cells (Supplementary Figure S5). These findings suggest that CD147 might be a broad-spectrum target for cancer therapy.

Monoclonal antibodies are advantageous for the treatment of cancer [31]. In order to achieve clinical therapeutic effects, antibody levels in serum must be high [32]. Adeno-associated virus vectors that transferred the anti-HIV mAb gene yielded low levels of antibody [33]. Recombinant oncolytic Newcastle Disease Virus carrying the full-length mouse-human chimeric HAb18 gene (cHAb18) induced HCC cell necrosis and prolonged survival in mice through inhibition of local tumor metastasis [34]. Here, we used the oncolytic adenoviral vector ZD55 to mediate HAb18 mAb expression in combination with the therapeutic cytokine IL24, which were linked by an F2A sequence. High expression of HAb18 and IL24 could still be observed 33 days after injection of ZD55-HAb18-IL24 into nude mice bearing PLC/PRF/5 xenograft tumors (Figure 5D and 5E). However, when the vector was injected into to C57/BL6 mice bearing syngeneic colon tumors, low serum HAb18 expression was observed. This could have resulted from defective replication of the oncolytic adenovirus in murine cells (Supplementary Figure S6).

Oncolytic adenoviral vectors can be generated using two strategies. One strategy is to use a tumor-specific promoter to drive an essential viral replication gene. Another strategy is to delete a viral element that is required for virus replication in normal cells but dispensable in tumor cells. The adenovirus E1B55KD enhances cyclin E expression and is required for efficient viral DNA replication [35]. E1B55KD deletion might attenuate oncolytic adenovirus replication in tumor cells. Luo et al. constructed the AdCN205-IL24 vector in which the hTERT promoter was used to control the expression of E1A in which the CR2 region was deleted. The expression of IL24 was controlled by the adenoviral E3 promoter. AdCN205-IL24 could express IL24 and suppress tumor growth [36].

Blocking HAb18G/CD147 using mAb HAb18 was shown to inhibit HCC growth *in vivo* [37]. Licartin (^131^I-labled HAb18) was approved for clinical use by the Food and Drug Administration of China in April 2005 and is an effective and safe treatment for HCC [38]. IL24 has been widely investigated because it has selective antitumor effects in HCC [36, 39, 40]. Ad-IL24 was effective in phase I/II clinical trials in patients with advanced cancers [41]. We hypothesized that the combination of the HAb18 and IL24 could result in enhanced antitumor effects. We demonstrated that oncolytic adenoviruses carrying both genes more effectively inhibited HCC cell growth than either gene alone both *in vitro* and *in vivo*. Furthermore, increases in apoptosis and autophagy were observed in ZD55-HAb18-IL24-treated cells (Figure 4 and 5). Collectively, our data indicate that ZD55-HAb18-IL24 has anti-tumor effects and is a potential therapeutic strategy for HCC.

## MATERIALS AND METHODS

### Patient samples

Adjacent, non-cancerous tissue samples were anonymously obtained from HCC patients treated at the Center of Clinical Oncology at the Affiliated Hospital of Xuzhou Medical College. All human samples were obtained with informed consent, and approval for usage was obtained from the Ethics Committee of the Affiliated Hospital of Xuzhou Medical College. Studies using these samples were approved by the Institutional Review Board of the Institute of Biochemistry and Cell Biology, Shanghai Institutes for Biological Sciences, Chinese Academy of Sciences.

### Cell culture

The human HCC cell lines (PLC/PRF/5, HepG2, BEL-7404, and Hep3B), human bladder cancer cell line (T24), human hepatocytes (QSG-7701), HEK-293T cells, and the murine colon cancer cell line (MC38) were purchased from the Cell Bank of the Type Culture Collection of the Chinese Academy of Sciences (Shanghai, China). The HEK-293 cell line was obtained from Microbix Biosystems Inc. (Toronto, Canada). All cells were cultured according to the manufacturer's instructions.

### Establishment of CD147-overexpressing and CD147-knockdown cells

Full-length CD147 cDNA was amplified by PCR and cloned into the lentiviral vector pLVX. The lentiviral stock was produced in 293T cells by transfecting pLVX-CD147 and the lentivirus backbone plasmids pMD2G and pSPAX2. Cells were infected with the lentivirus vectors and selected with puromycin (10 μg/mL). For stable knockdown, shRNA against CD147 (5′-GGTTCTTCGTGAGTTCCTC-3′, 5′-GTACAAGATCACTGACTCT-3′) and a scramble control shRNA (5′-CTACCGTTGTTATAGGTG-3′) were synthesized and cloned into the pLVX vector.

### Adenovirus construction

The GFP, IL24, HAb18, and HAb18-F2A-IL24 expression cassettes were inserted into pShuttle-E1B (Δ55) with E1B55K deletion. Oncolytic adenovirus plasmids were generated by homologous recombination of the shuttle vector and the adenoviral backbone plasmid in *E. coli* BJ5183 cells. Oncolytic adenoviruses were packaged and amplified in HEK-293 cells, and then purified by gradient CsCl centrifugation. Virus titer was measured using the Quick Titer Adenovirus Titer Immunoassay Kit (Cell Biolabs, San Diego, CA, USA), or plaque assays (HEK293 cells). Viral genomic DNA was extracted using the Blood Genome Extract Kit and the manufacturer's protocol (Generay, Shanghai, China) for identification. The absence of wild-type contamination was demonstrated by PCR with corresponding primers (Supplementary Table S1). The F2A sequence was generated from the FMDV 2A sequence through the addition of a furin cleavage site sequence (RAKR) to the N-terminus of the FMDV 2A sequence.

### Quantitative RT-PCR

Total RNA was isolated using TRIzol (CWBIO, Beijing, China). Single-strand cDNA was synthesized using the ReverTra Ace qPCR RT Kit (Toyobo, Osaka, Japan). RNA expression was analyzed using SuperReal Premix Plus (TIANGEN, Beijing, China) according to the manufacturer's protocols. The sequences of all primers are shown in Supplementary Table S1.

### Western blotting

Protein concentrations were estimated using the Lowry assay (BioRad, Hercules, CA, USA). Western blotting was performed using standard protocols and the following antibodies: anti-procaspase-9, -procaspase-3, -PARP, -GFP (Santa Cruz biotechnology, Santa Cruz, CA, USA), -IL24 (GenHunter Corporation, Nashville, TN, USA), -HA, -actin, -GAPDH (CWBIO, Beijing, China), -LC3, -p62, and –beclin-1 (Sigma, St. Louis, MO, USA). All HRP-conjugated secondary antibodies were purchased from Santa Cruz Biotechnology.

### Proliferation assays

Cell proliferation was measured using the CCK-8 kit (Dojindo, Kumamoto, Japan). Following treatment, the cells were incubated with 10 μL CCK-8 at 37°C for 2 h. Absorbance was measured at 450 nm and 630 nm using a Biotek Eon Microplate Reader.

### Migration assays

Cell migration assays were performed using transwell inserts (8 μm, BD Biosciences, San Jose, CA, USA) placed in 24-well plates. Cells were cultured in the upper chambers of the transwell inserts in 200 μL serum-free medium. The medium in the bottom chamber contained 10% fetal bovine serum. Following a 12 h incubation, the inserts were stained with 0.5% crystal violet and imaged.

### Flow cytometry

Apoptosis was analyzed using the Annexin V-FITC Apoptosis Detection kit (Beyotime Biotechnology, Shanghai, China) according to the manufacturer's instructions. The mitochondrial membrane potential was evaluated by staining the cells with the JC-1 fluorescent probe (BD Biosciences) and then subjecting them to flow cytometry using a FACS Calibur flow cytometer (BD Biosciences).

### Immunofluorescence microscopy

Cells were fixed with 4% (w/v) paraformaldehyde, permeabilized using 0.1% (w/v) Triton X-100 and blocked with 1% bovine serum albumin. The cells were stained with an anti-LC3 antibody (Sigma) overnight and then incubated with a Cy3-labeled secondary antibody in PBS with 1% FBS for 1 hour at room temperature. Images were captured using a laser scanning confocal microscope (Olympus, Tokyo, Japan).

### Animal experiments

All *in vivo* experiments were performed according to protocols approved by the U.S. Public Health Service Policy on Humane Care and Use of Laboratory Animals. Four-week-old female BALB/c nude mice and C57/BL6 mice were purchased from the Animal Core Facility (Shanghai, China). We pre-mixed 5 × 10^6^ PLC/PRF/5 cells with Matrigel (BD Biosciences) at a 1:1 ratio and subcutaneously injected the cells into the right flank of each mouse. A total of 1 × 10^6^ MC38 cells were injected into C57/BL6 mice. When the tumors reached 80–120 mm^3^, the mice were randomly divided into five groups (six mice per group). Oncolytic adenovirus (2.5 × 10^8^ PFU per mouse) or PBS was intratumorally injected every other day (four injections total). The tumor volume was measured using a Vernier caliper every 4 days and calculated using the following equation: (length × width ^2^) / 2. At the end of the experiment, the tumors were resected from the sacrificed mice for immunohistochemical analysis. The levels of AST, ALT, creatinine, and BUN in serum were measured.

### ELISA

Serum HAb18 expression was quantified by ELISA using a human IgG ELISA detection kit (Mlbio, Shanghai, China). All experiments were performed according to the manufacturer's protocols.

### Immunohistochemistry and TUNEL assays

Tumor tissue was fixed in 4% formaldehyde overnight, embedded in paraffin, and sectioned (5 μm thickness) for immunohistochemical analysis. Sections were stained with an anti-CD31 antibody (Cell Signaling Technology, Danvers, MA, USA). TUNEL assays were performed according to the manufacturer's protocol (Roche, Basel, Switzerland). Hematoxylin was used as a counterstain.

### Transmission electron microscopy

Tumor tissue was fixed with 2.5% glutaraldehyde overnight followed by 1% osmium tetroxide for 1.5 h. The tissue samples were then dehydrated using a graded series of ethanol. Samples were then rinsed with acetone and permeated overnight with embedding buffer. Sections of 70 nm thickness were dual-stained with 2% uranyl acetate and lead citrate. Autophagosomes were examined by transmission electron microscopy (FEI, Hillsboro, OR, USA).

### Statistical analysis

Data are presented as the mean ± standard deviation (S.D). Comparisons between two groups were performed with Student's t-tests and the GraphPad Prism 6.0 software (GraphPad Software, USA).

## SUPPLEMENTARY FIGURES AND TABLE


