# Supplementary material for: An oncolytic adenovirus that expresses the HAb18 and interleukin 24 genes exhibits enhanced antitumor activity in hepatocellular carcinoma cells
Source: Oncotarget. 2016 Aug 9;7(37):60491–502. doi: 10.18632/oncotarget.11134 (PMC5312398; doi:10.18632/oncotarget.11134)
Supplement: Supplementary file 1 [file oncotarget-07-60491-s001.pdf]

An oncolytic adenovirus that expresses the HAb18 and interleukin 24 genes exhibits enhanced antitumor activity in hepatocellular carcinoma cells

SUPPLEMENTARY FIGURES AND TABLE

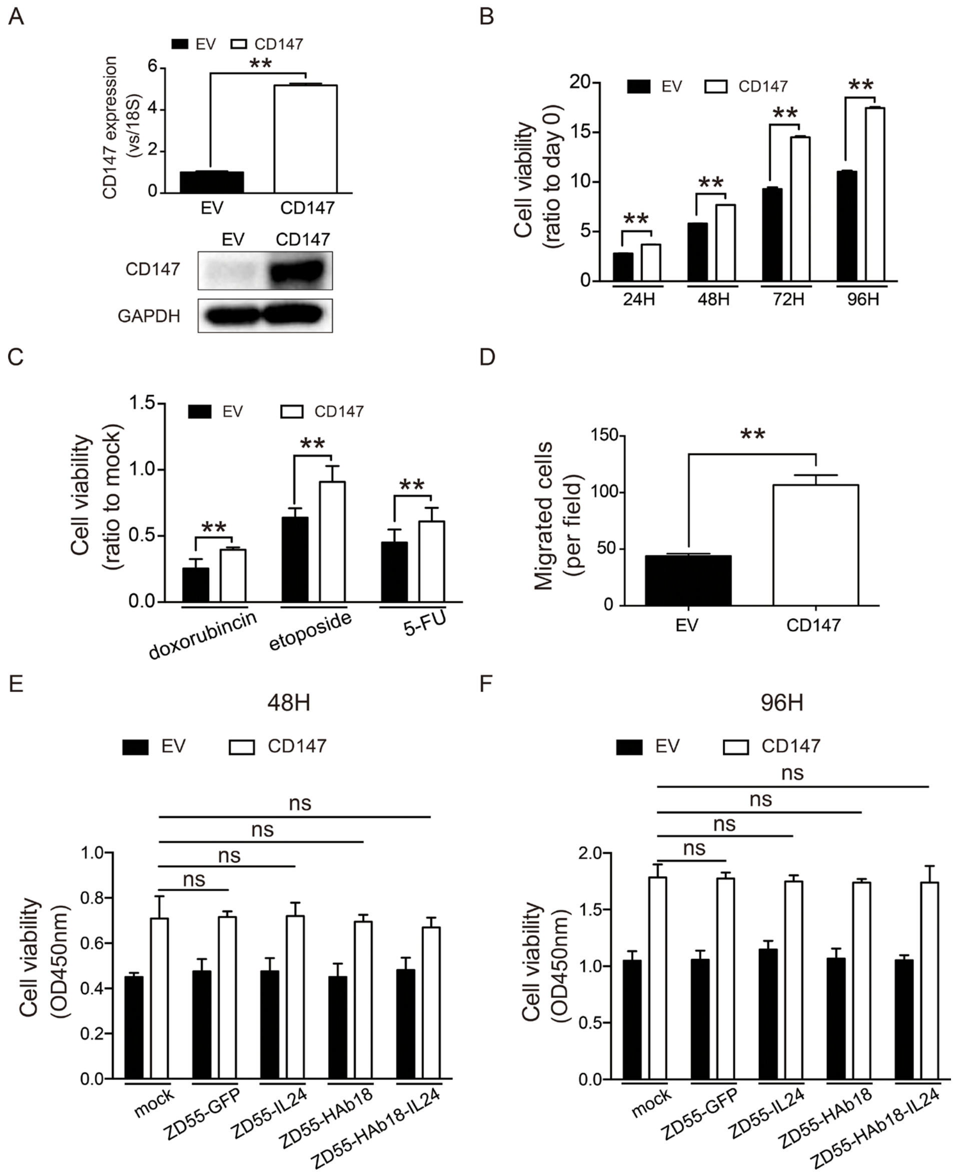

Supplementary Figure S1: Oncolytic adenoviruses do not suppress the growth of CD147-transduced QSG-7701 cells. **A.** Overexpression of CD147 in QSG-7701 cells was confirmed by qRT-PCR and western blot. The qRT-PCR data were normalized to 18S and are shown as the fold change relative to QSG-7701 cells in the EV groups. GAPDH was used as a loading control in the western blot analysis. **B.** CD147 overexpression enhances QSG-7701 cell viability. **C.** CD147 overexpression increases the resistance of QSG-7701 cells to cytotoxic chemotherapy. Cells were treated with doxorubicin (1 μg/mL), etoposide (10 μg/mL), or 5-FU (100 μg/mL) for 2 days. Relative cell viability is shown as the fold change compared to corresponding mock cells. **D.** Overexpression of CD147 enhanced QSG-7701 cell migration. Cell migration was analyzed using transwell assays. Five random fields per treatment were counted. **E, F.** The viability of CD147-overexpressing QSG-7701 cells infected with the indicated oncolytic adenoviruses at an MOI of 10 for 48 (E) and 96 hours (F). Cell viability was measured using CCK-8 assays. All experiments were repeated three times. The bars represent the mean ± S.D. (n = 3), $^{**}p < 0.01$, ns, no significance. EV, empty vector.

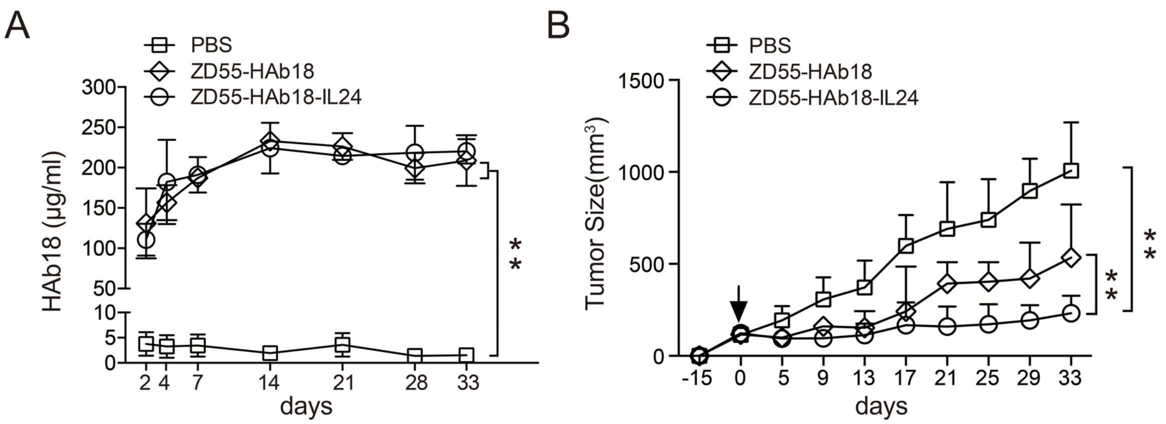

Supplementary Figure S2: The kinetics of HAb18 expression in the serum of oncolytic adenovirus-treated mice. Oncolytic adenoviruses were intratumorally injected after subcutaneous inoculation of PLC/PRF/5 cells (day 0, arrow). Corresponding volumes of PBS were injected as a control. **A.** Detection of HAb18 in serum from treated mice by ELISA. **B.** Growth curve of PLC/PRF/5 xenograft tumors after intratumoral injection of the indicated adenoviruses. The bars represent the mean ± S.D. ($n = 6$), $^{**}p < 0.01$.

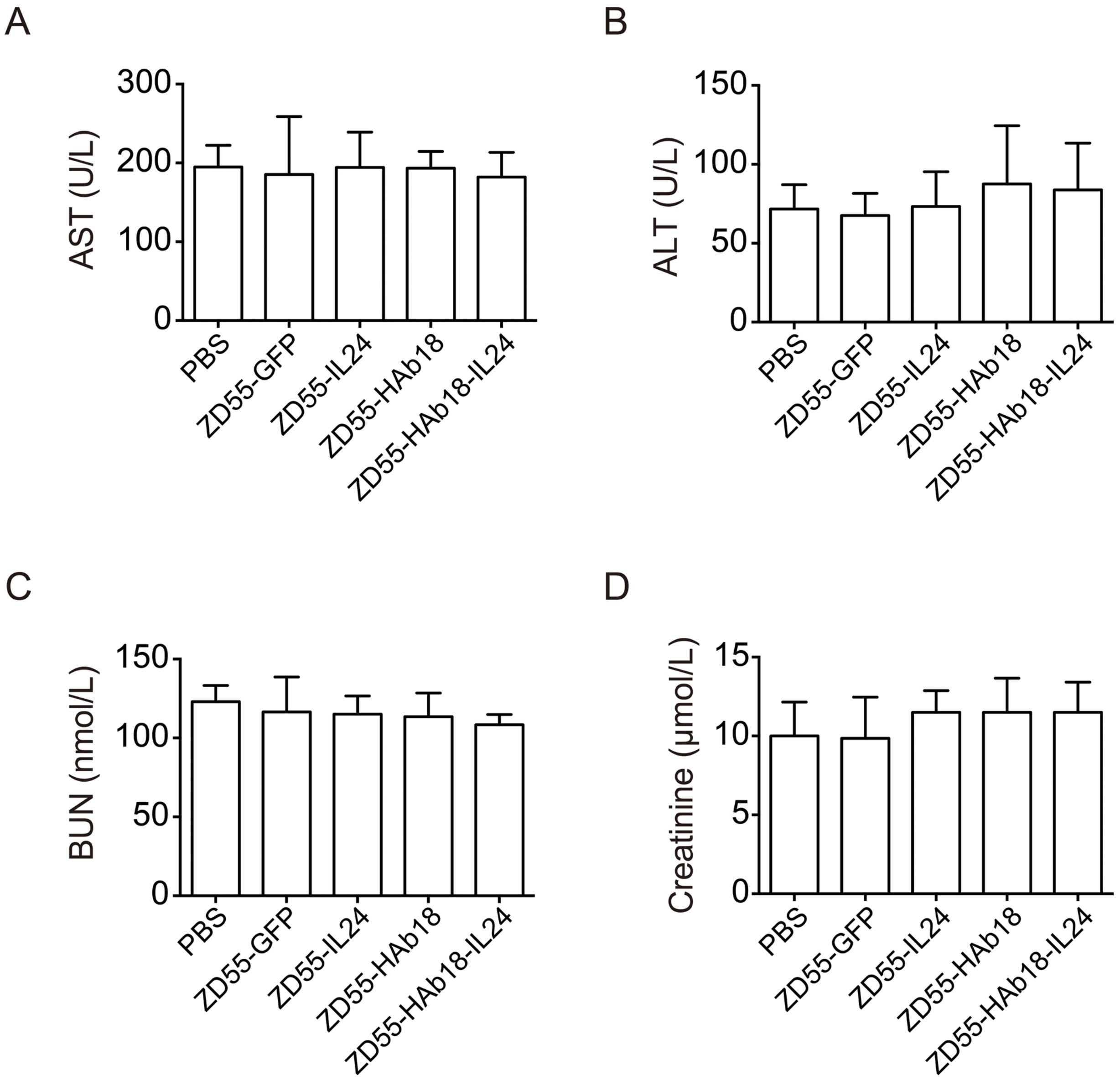

Supplementary Figure S3: Assessment of hepatotoxicity and renal toxicity caused by oncolytic adenoviruses. Serum AST, ALT, BUN, and creatinine were measured after the mice were sacrificed. AST, aspartate aminotransferase; ALT, alanine aminotransferase; BUN, blood urea nitrogen. The bars represent the mean ± S.D. (n = 6).

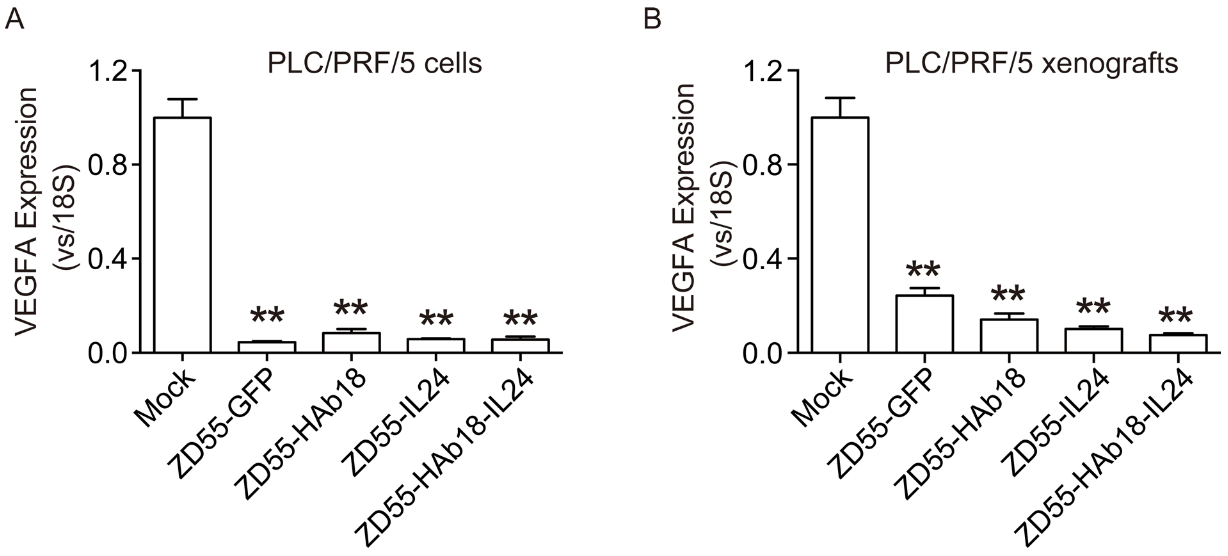

Supplementary Figure S4: ZD55-HAb18-IL24 treatment decreased the mRNA level of VEGFA in PLC/PRF/5 cells. **A.** PLC/PRF/5 cells were infected with the indicated oncolytic adenoviruses (MOI of 10) for 2 days and the expression of human VEGFA measured by qRT-PCR. **B.** The expression of human VEGFA in tumors from each group was measured by qRT-PCR. Data were normalized to 18S, and are shown as the fold change relative to mock cells. All experiments were repeated three times. Bars represented mean ± S.D. (n = 3). **$p < 0.01$.

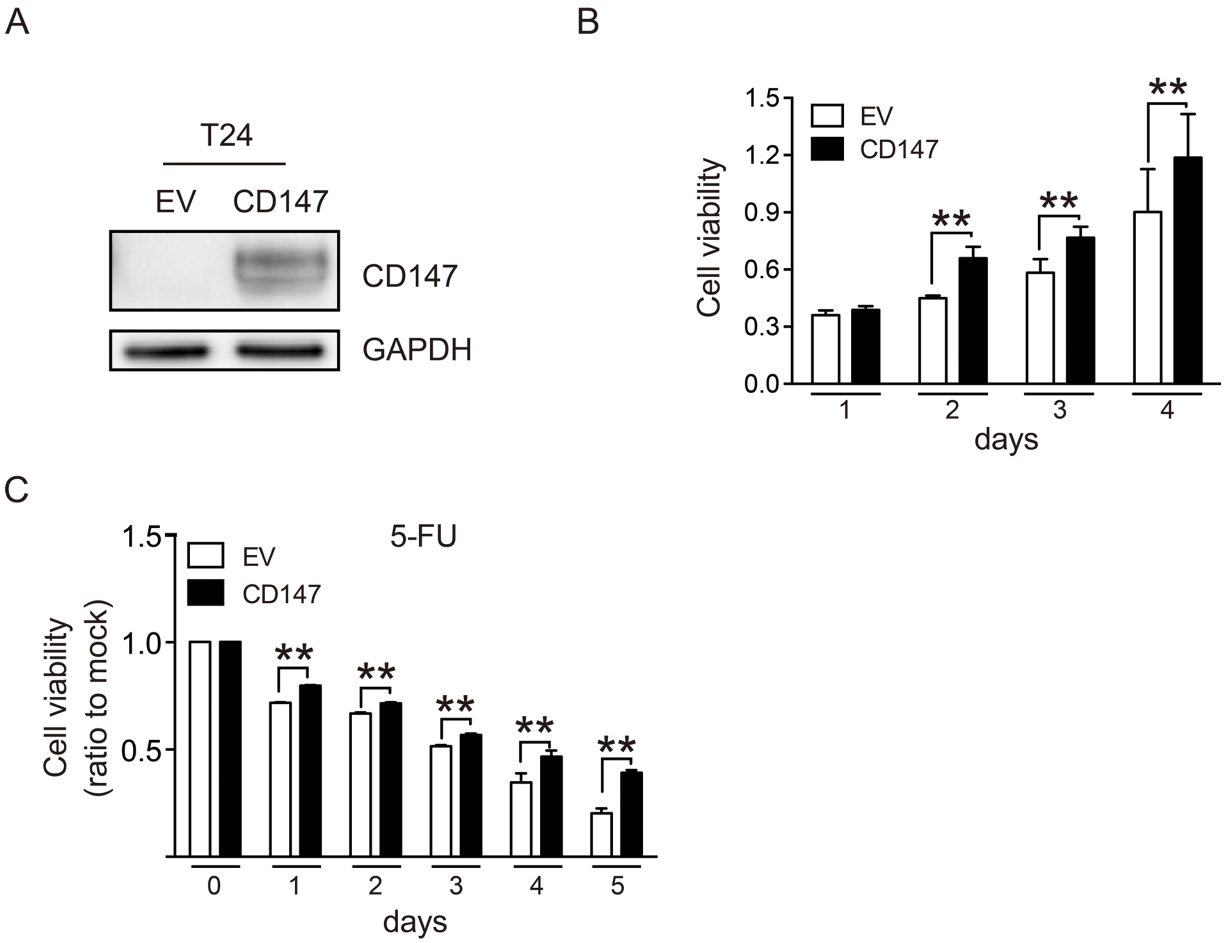

Supplementary Figure S5: CD147 overexpression promotes T24 cell proliferation and 5-FU resistance. A. CD147 overexpression was confirmed by western blot. GAPDH was used as a loading control. **B.** Overexpression of CD147 promotes T24 cell proliferation. **C.** Overexpression of CD147 enhances 5-FU resistance in T24 cells. Cells were treated with 5-FU (100 μg/mL) for 2 days. The relative cell viability is shown as the fold change compared to corresponding mock cells. Cell viability was analyzed using CCK-8 assays. EV, empty vector. All experiments were repeated three times. The bars represent the mean ± S.D. (n = 3). **p < 0.01.

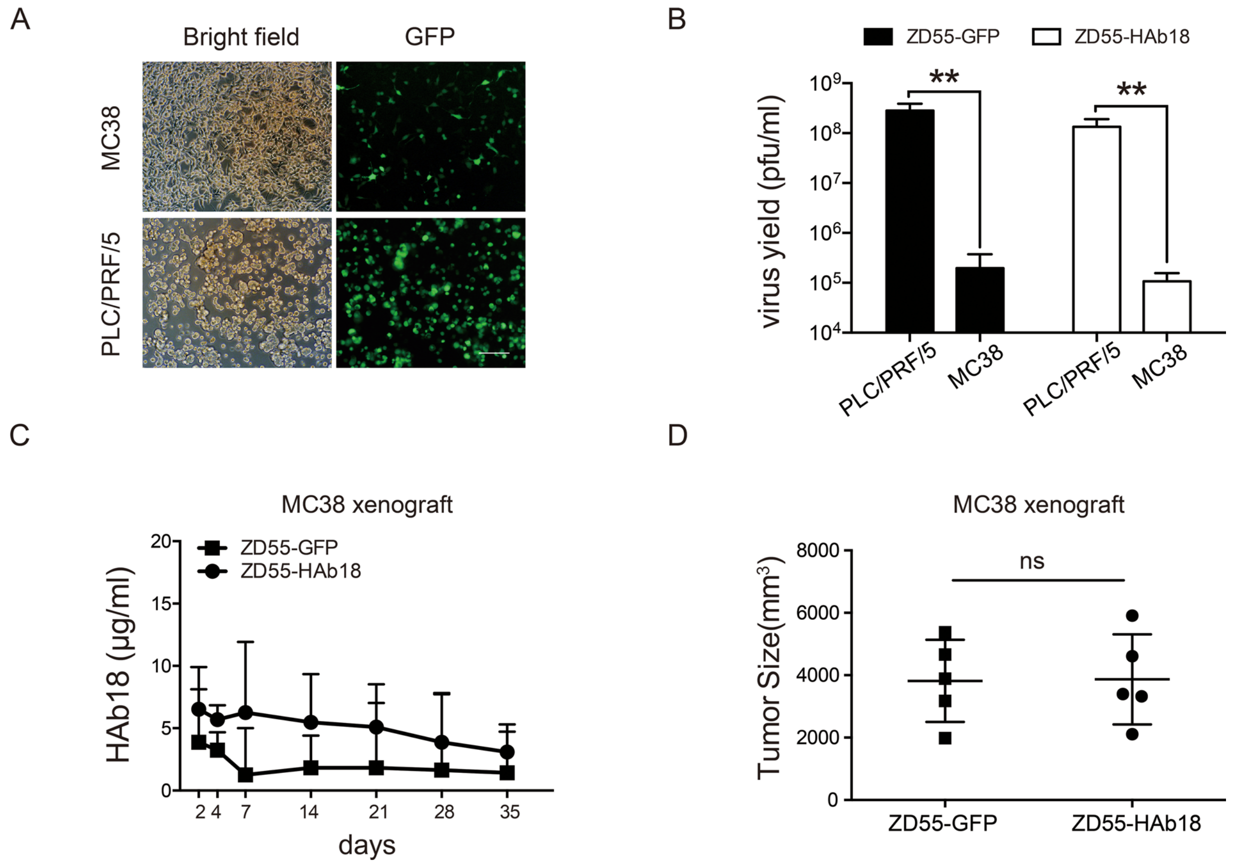

Supplementary Figure S6: ZD55-HAb18 does not efficiently express HAb18 in MC38 murine colon cancer cells. A. Representative images of MC38 and PLC/PRF/5 cells infected with ZD55-GFP for 48 hours. Scale bar, 100 µm. **B.** MC38 and PLC/PRF/5 cells were infected with ZD55-GFP and ZD55-HAb18 at an MOI of 10 for 48 hours. The capacity of viral replication was assessed using viral progeny assays. Experiments were repeated three times. **C.** HAb18 concentrations in the serum of mice treated with the viruses at the indicated time points after injection measured by ELISA. **D.** Volumes of MC38 xenografts treated with ZD55-GFP and ZD55-HAb18 35 days after injection. The bars represent the mean ± S.D. (n = 3 [B], n = 5 [C, D]). **p < 0.01, ns, no significance.

Supplementary Table S1: Nucleotide sequence of oligonucleotide primers used for virus identification and RT-PCR

Gene	Direction	Nucleotide sequence
E1B-55KD	forward<br>reverse	5'-TTGACAATTACAGAGGAT-3'<br>5'-GTAGGATAAGGTTGGTAT-3'
CD147	forward<br>reverse	5'-ACTCCTCACCTGCTCCTTGA-3'<br>5'-GCCTCCATGTTCAGGTTCTC-3'
MMP-2	forward<br>reverse	5'-GGCAGTGCAATACCTGAACACC-3'<br>5'-GTCTGGGGCAGTCCAAAGAACT-3'
E1A	forward<br>reverse	5'-TGCAGGTCTTGTCATTATCAC-3'<br>5'-ATGCCACAAGGTCCTCATATAG-3'
MMP-9	forward<br>reverse	5'-TTCCCCTTCACTTTCCTGGGTA-3'<br>5'-CGCCACGAGGAACAAACTGTAT-3'
HAb18	forward<br>reverse	5'-AACTGCTGATATTCTATG-3'<br>5'-GAATGGAGGACTATAATC-3'
IL24	forward<br>reverse	5'-CACAATAGAACAGTTGAAGTC-3'<br>5'-GTGACACGATGAGAACAA-3'
VEGFA	forward<br>reverse	5'-ATTATGCGGATCAAACCT-3'<br>5'-TTCTTGTCTTGCTCTATCTT-3'
18S	forward<br>reverse	5'-AACTTTCGATGGTAGTCGCCG-3'<br>5'-CCTTGGATGTGGTAGCCGTTT-3'
